# The non-destructive investigation of a late antique knob bow fibula (Bügelknopffibel) from Kaiseraugst/CH using Muon Induced X-ray Emission (MIXE)

**DOI:** 10.1186/s40494-023-00880-0

**Published:** 2023-03-02

**Authors:** Sayani Biswas, Isabel Megatli-Niebel, Lilian Raselli, Ronald Simke, Thomas Elias Cocolios, Nilesh Deokar, Matthias Elender, Lars Gerchow, Herbert Hess, Rustem Khasanov, Andreas Knecht, Hubertus Luetkens, Kazuhiko Ninomiya, Angela Papa, Thomas Prokscha, Peter Reiter, Akira Sato, Nathal Severijns, Toni Shiroka, Michael Seidlitz, Stergiani Marina Vogiatzi, Chennan Wang, Frederik Wauters, Nigel Warr, Alex Amato

**Affiliations:** 1 Paul Scherrer Institute PSI, 5232 Villigen, Switzerland; 2 Archäologisches Institut, Universität zu Köln, Albertus-Magnus-Platz, 50923 Köln, Germany; 3 Augusta Raurica, Schwarzackerstrasse 2, 4302 Augst, Switzerland; 4 KU Leuven, Instituut voor Kern-en Stralingfysica, 3001 Leuven, Belgium; 5 Institut für Kernphysik, Universität zu Köln, Zülpicher Strasse 77, 50937 Köln, Germany; 6 Graduate School of Science, Osaka University, Toyonaka, Osaka Japan; 7 Departimento di Fisica, Università di Pisa and INFN sez. Pisa, Largo B. Pontecorvo 3, 56127 Pisa, Italy; 8 Institute for Particle Physics and Astrophysics, ETH Zürich, 8093 Zürich, Switzerland; 9 PRISMA+ Cluster of Excellence and Institute of Nuclear Physics, Johannes Gutenberg Universität Mainz, 55128 Mainz, Germany

**Keywords:** Knob bow fibula (Leutkirch type), Copper alloy, Muon Induced X-ray Emission (MIXE), Non-destructive elemental analysis, Depth-resolved

## Abstract

A *knob bow fibula (Bügelknopffibel)* of the *Leutkirch* type, which typologically belongs to the second half of the 4th and early 5th century CE, was excavated in 2018 in the Roman city of Augusta Raurica, present-day Kaiseraugst (AG, Switzerland). This was analyzed for the first time for its elemental composition by using the non-destructive technique of Muon Induced X-ray Emission (MIXE) in the continuous muon beam facility at the Paul Scherrer Institute (PSI). In the present work, the detection limit is 0.4 wt% with $\sim$1.5 hours of measurement time. The fibula was measured at six different positions, at a depth of 0.3–0.4 mm inside the material. The experimental results show that the fibula is made of bronze, containing the main elements copper (Cu), zinc (Zn), tin (Sn) and lead (Pb). The compositional similarities/differences between different parts of the fibula reveal that it was manufactured as two “workpieces”. One workpiece consists of the knob (13.0±0.6 wt% Pb), bow (11.9±0.4 wt% Pb) and foot (12.5 ± 0.9 wt% Pb). These show a higher Pb content, suggesting a cast bronze. The spiral (3.2 ± 0.2 wt% Pb), which is part of the other workpiece, has a comparatively lower Pb content, suggesting a forged bronze.

## Introduction

Archaeological objects allow us to shed light on past societies. They can be used to identify social practices that are important for understanding past cultures. Through scientific methods of analysis, the possibilities of the interpretation of archaeological objects have continuously grown over time. Whereas until a few decades ago stylistic features were the only criteria available, nowadays physico-chemical analyses complement the distinguishing criteria in many respects. The stylistic features are by no means to be minimized. On the contrary, they continue to be the starting point for all further investigations and considerations. In this context, an ideal route is the application of the Muon Induced X-ray Emission (MIXE)^1^ analysis technique, which allows a completely non-destructive elemental determination without detectable radiation damage, and which can measure up to a few centimeters (cm) inside the material due to its high-energy muon induced X-rays.

Already at the end of the 1990s, J. Lutz pointed out the fact that all non-destructive analysis methods (with the exception of very small objects, which can be measured with Neutron Activation Analysis (NAA), that also provides the bulk properties) suffer from the fact that only the object’s surface can be measured [10]. Actually, in contrast to electron-induced X-ray analysis techniques, the MIXE technique can provide results below the usually distorted surface of archaeological metal objects. Examples of applications of this analysis technique to archaeological metal objects are already available [4, 6–9, 11–13], all of which were performed at pulsed muon beam facilities. It was also shown recently [1, 2] that the unique experimental setup at the Paul Scherrer Institute (PSI), which utilizes continuous muon beams, enables one to achieve high statistics measurements in short measurement periods.

Our interdisciplinary research project offers for the first time an elemental analysis of a knob bow fibula, excavated in 2018, near the Late Roman fort at Kaiseraugst. This late antique fibula type was widespread especially in large parts of the Germania libera. In Roman contexts, they are rather rarely found, which is why it can be assumed that single individuals were brought into the working area of Roman Switzerland. Because of this special position and the good conservation status a destructive analysis of the fibula discussed here was excluded. Thus, it was examined non-destructively for its elemental composition using the MIXE technique. In addition to validating the applicability of the MIXE technique for cultural artefacts, the aim of the investigation was to take a detailed look at the manufacturing method used and composition of the archaeological object.

## The Knob bow fibula from Augusta Raurica: past findings

**Fig. 1:**
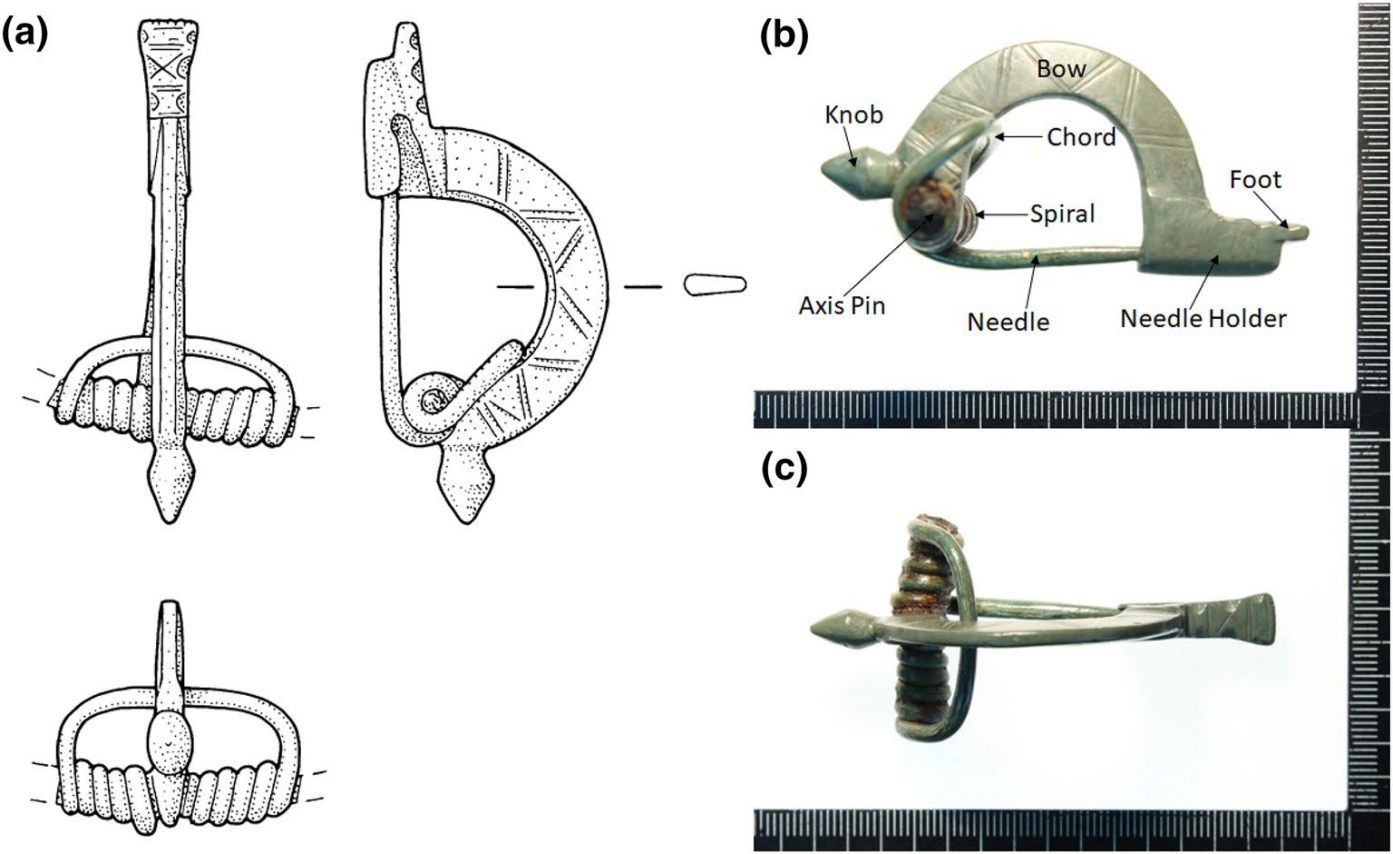
**a** Schematic drawings of the *knob bow fibula* (*Bügelknopffibel* with inventory number 2018.005.G07151.2) from different angles (drawing: C. Stierli).** b** and **c** show the actual photographs of the same fibula from different viewpoints. The different parts of the *fibula* are labeled in **b**

**Fig. 2:**
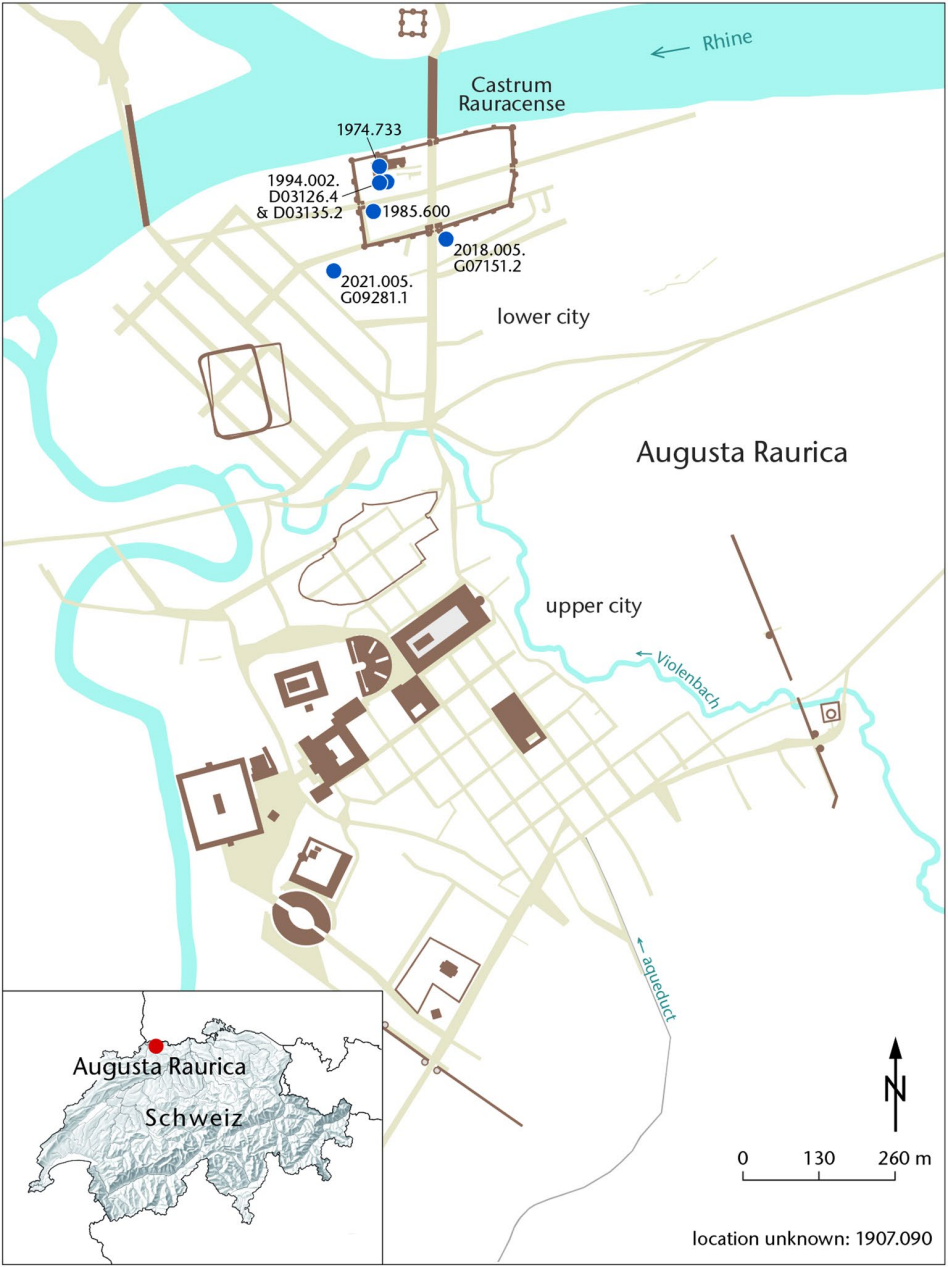
A schematic map of the ancient Roman city of Augusta Raurica, showing the most important streets and monumental buildings of the Imperial period. The Late Roman fortress called Castrum Rauracense, located near the river Rhine, is shown. Findspots of the different *knob bow fibulae* (including the one mentioned here) are shown by blue filled circles, along with their inventory numbers. The inset at the bottom left-hand corner shows the map of Switzerland with the location of Augusta Raurica marked by a red filled circle. (map: C. Zipfel)

### Excavation site

The object, shown in Fig. 1, with inventory number 2018.005.G07151.2 was excavated in 2018 in the Roman city of Augusta Raurica, present-day Kaiseraugst (AG, Switzerland), and published in 2019 [14, p. 107]. The excavation was prompted by a planned construction project. A map of the Roman city of Augusta Raurica is shown in Fig. 2. The excavation area is located in the eastern lower city of Augusta Raurica, directly adjacent to the east of the so-called Castrumstrasse [14, p. 95]. In late antiquity, two defensive ditches ran in front of the Castrum Rauracense (shown in Fig. 2). This fibula was found in the backfill of the earlier weir ditch. The relative dating of the excavation of the earlier weir ditch is not before the 5th century CE, according to the excavators [14, p. 111].

### Description and manufacturing

**Fig. 3:**
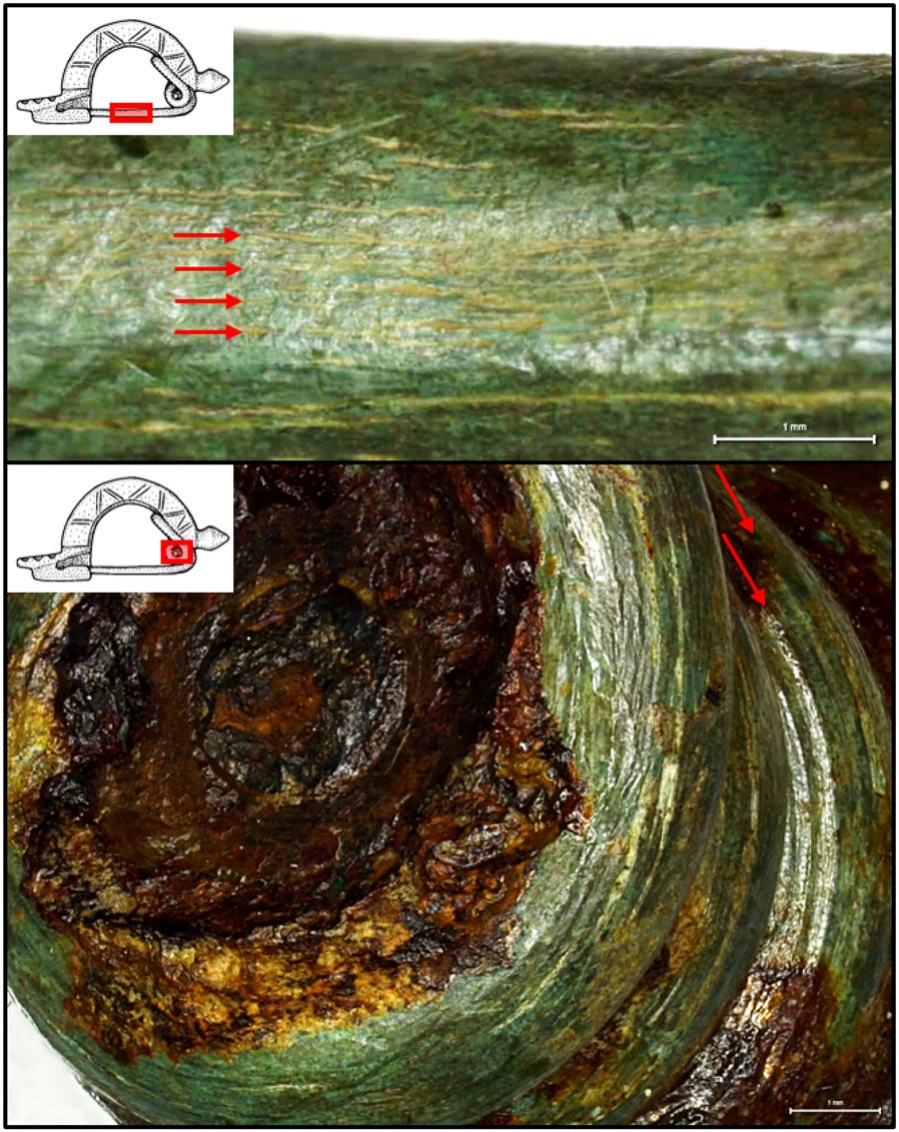
Detailed photos of the needle (top) and the spiral (bottom) of the fibula. The photos were taken with a Leica DVM6 stereomicroscope. The arrows indicate the manufacturing traces. The corrosion products of the iron (from the axis-pin) are clearly visible on the spiral

The nomenclature of the different parts of the fibula is depicted in Fig. 1b. The fibula, made of non-ferrous metal, is complete and in a good state of preservation. The surface is completely covered with an olive green patina. Only on the spiral some reddish-brown corrosion products are visible, which suggests an iron axis-pin. The fibula was uncovered using various mechanical tools and a protective coating of low-viscosity acrylic resin at 5% concentration was applied (Restoration and Conservation Christine Pugin, 2018–2019). After completion of the conservation-restoration measures, the fibula weighs 33.53 g and has a length of 6.50 cm. At the widest point, it measures 3.20 cm and is a maximum of 3.30 cm high. Based on the dimensions, the method of manufacture (cast vs. forged) can usually be reconstructed, although an absolute delimitation is not always possible [15, p. 455–456]. For the fibula corpus, it can be assumed that the workpiece was cast. The cross-sectionally highly trapezoidal bow is semicircularly arched up and decorated with (double) grooves (cf. bow decoration of the knob bow fibula from Heilbronn with simple grooves [16, p. 44, Fig. 13.6]). The bow bends triangularly mediated into a straight foot. This is also decorated with a pattern of three semicircular notches on each side with transverse and cross grooves on the surface. The notches show a round profile, which is why chasing by means of hammer and punch can be concluded. The foot widens slightly starting from the bow and finishes in a slight wave shape. The fibula has a double conical knob that emerges straight from the bail and is slightly faceted. The bow and the knob show grooves and a slight faceting, respectively, due to a modest surface reworking. The moderately long spiral construction with high lower chord consists of ten turns. It is no longer possible to determine whether a finial in the form of buttons or simple caps sat on the ends of the spiral construction. The needle holder, which is fixed on the right and closed at the bottom, but has a small hole at the end, is box-shaped and occupies almost the entire length of the foot. The needle holder offers an important indication of the manufacturing method of fibulae. The characteristic feature of a cast needle holder on an unfinished cast fibulae is the often stronger, often even widening cross-section in contrast to the pointed triangular cross-section of forged needle holders. In addition, it has a thickness of usually over 1.2 mm just above the needle notch [15, p. 458, Fig. 6]. In the case of this fibula, the thickness of the cast needle holder is 1.9 mm. The fibula is constructed in two parts: (i) The needle holder, foot, bow and knob consist of a solid cast workpiece. The bow is perforated at the head and receives the axis-pin around which the spiral is wound and (ii) the spiral, chord and needle are made from a different workpiece. The diameter and length of the needle determine the closing force of the fibula, i.e., the longer the needle, the larger its diameter must be to have the same closing force. For this fibula, the needle has a length of 34 mm and a diameter of 3 mm. Knob bow fibulae have a comparatively high closing force [15, p. 459, Fig. 9], which makes their use as coat clasps very conceivable. Fabrication marks in the form of longitudinal grooves are visible on the needle and the spiral due to wire drawing or hammering (see Fig. 3). The needle structure is subjected to the greatest mechanical stress, which is why a forgeable alloy, rather than a cast material, was normally used here. It is important to note that forged wires are more resilient than drawn wires, where tensile stresses prevail. Parallel longitudinal grooves such as those seen here do not necessarily indicate wire drawing, but can also occur when a forged wire is rounded and smoothed. It is also possible that the forged wire was drawn through a drawing hole to bring it into shape, not to lengthen it and reduce its cross-section. The spiral constructions of Germanic fibulae show different wire cross-sections, with oval or round cross-sections, as in this fibula, being considered a quality feature [15, pp. 458–459].

### Typology

The fibula, shown in Fig. 1, is a knob bow fibula (*Bügelknopffibel*) of the Leutkirch type. Knob bow fibulae are characterized by a wide range of variation. In the specialist literature, they are differentiated based on their various designs—primarily the knob, but also the other designs of the bow, foot, and needle holder [17, p. 47]. Knob bow fibulae are mostly made of bronze, sometimes with surface finishes, and have a spiral axis-pin made of iron. The bow is usually longer than the foot. The spiral, which is often wide, is made as a crossbow construction ([18, pp. 669–672]; [16, p. 42]). Most frequently represented are knob bow fibulae with polyhedral knob bows. But also double conical knobs, as in the case of this fibula, occur relatively often [19, pp. 216–226]. Under the type Leutkirch, R. Koch summarizes large brooches (up to 10 cm), which have a smooth, regular double conical, unstemmed knob, but also match each other in shape and proportion of the bow and foot [20, pp. 236–237]. The eponymous find is the knob bow fibula from an Alamannic male grave from Leutkirch/Baden-Württemberg [21, p. 116, pl. 21, Fig. 1]. A compilation of the knob bow fibulae of the Leutkirch type including distribution maps can be found in [22, p. 502, Fig. 14] and [16, p. 45, Fig. 14]. The closest spatial equivalents to the fibula analyzed here are from Jestetten/Baden-Württemberg [23, pp. 289–290, Fig. 72.1], Vindonissa (Windisch, AG/Switzerland) [16, pp. 187–188], and Hüttwilen (TG/Switzerland) [24, pp. 143–144, 149].

### Dating and distribution

Knob bow fibulae (*Bügelknopffibel*) are considered the Germanic counterpart of the Roman onion-knob fibulae (*Zwiebelknopffibel*) characteristic of Late Antiquity ([25, p. 84]; [26, pp. 562, 593]; [27, pp. 86–87]). They appear simultaneously with the onion-knob fibulae and seem to be related to or mutually influenced by it. Because of their chronology, they are not dependent on it, as is often assumed [19, pp. 242–245]. According to Meyer, they go back to the crossbow fibulae, which were widespread in Roman forts in the first half of the 3rd century CE, whereby a common origin could be assumed for them and the onion-knob fibulae. Knob bow fibulae have a long-term and wide distribution within the whole Germania libera from the end of the 3rd century until the beginning of the 6th century ([19, p. 254]; [28, p. 272]). They were indigenous (Germanic, non-Roman) products. According to Meyer, their provincializing character makes the Roman cultural influence in the Germania libera very clear [19, pp. 254–255]. In Roman contexts, only individual pieces are attested. From the (surroundings of the) Castrum Rauracense, including the fibula mentioned in this manuscript, six—among them a new find from 2021—knob bow brooches are currently known (see Fig. 2) ([25, p. 84, cat. no. 289–290]; [29, p. 75, cat. no. 2000]; [30, p. 71, 77, Fig. 14]; [31]). They make up only a fraction of the fibula types in the area of today’s Augst and Kaiseraugst. The distribution of small finds can have different reasons. It can possibly indicate the working area or the sphere of activity of a mobile craftsman, or the trade across tribal areas or, especially in this working area, the lively cross-border exchange. Of course, in the case of single finds away from the main distribution area, the mobility of the wearer, for example through military service, exogamy or travel, must also be considered. Possibly, the knob bow brooches found in the working area of northwestern Switzerland are pieces brought by Germanic mercenaries. Hoeper writes of a total of 20—including this fibula now 21—specimens that can be assigned to the Leutkirch type and are found in the Elbe region and mainly in southwestern Germany and northern Switzerland ([16, p. 45, Fig. 14]; [17, p. 47]; [20, p. 239, Fig. 7]). Local knob bow fibula production—especially of the Leutkirch type—is also assumed for southwestern Germany ([16, p. 43]; [32, p. 299]). However, due to the lack of casting moulds and cast-like pieces, no exact places of production can be determined so far. They are mostly, but not exclusively, found in men’s graves and seem to have taken their origin as coat clasps in a military context ([33, p. 285]; [32, pp. 290–291]; [22, p. 446]; [16, p. 43]; [28, p. 272]). The Leutkirch type dates to the second half of the 4th century and the early 5th century ([34, p. 31]; [28, p. 272]; [22, p. 501]; [16, pp. 46–47]).

## Experimental details

**Fig. 4:**
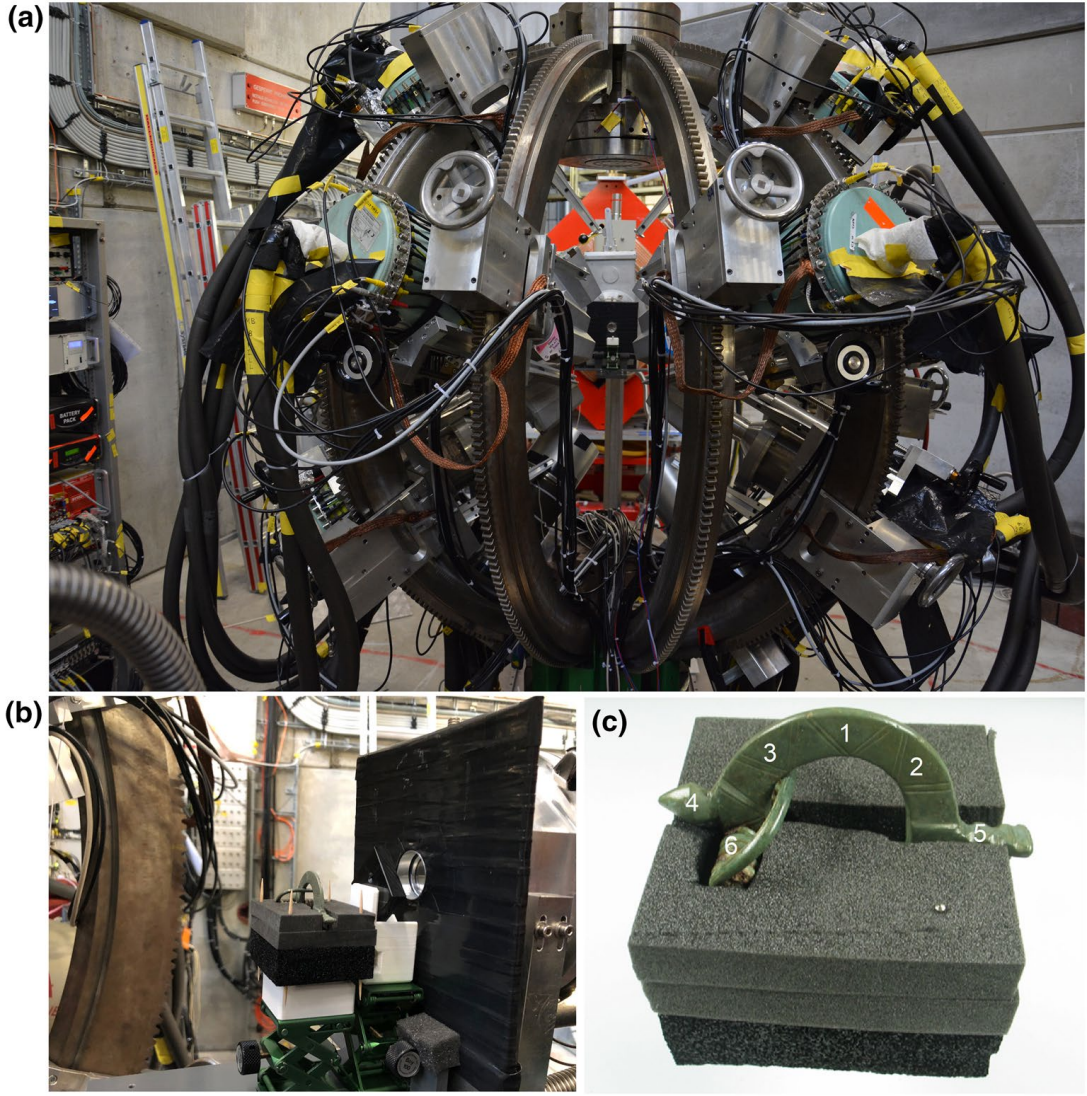
**a** The MINIBALL Germanium detector array. **b** The positioning of a plastic collimator and the knob bow fibula. **c** The six different positions of the knob bow fibula (mounted on polyethylene foam), measured during the experiment (see text in Sect. "Measurement of the knob bow fibula (Bügelknopffibel)" for explanation on the different positions)

### Experimental setup

All the measurements described in the following subsections were performed using the Muon Induced X-ray Emission (MIXE) technique at the Paul Scherrer Institute (PSI), in the $\pi$E1 experimental area. The High Intensity Proton Accelerator (HIPA) complex at PSI consists of a cascade of three accelerators, resulting in a final proton beam energy of 590 MeV at a current of up to 2.4 mA [35]. This proton beam passes through a target made of carbon where the deposited energy creates numerous secondary particles, among them so called pions ($\pi ^{+}$ and $\pi ^{-}$), which in turn decay into muons ($\mu ^{+}$ and $\mu ^{-}$) within several nanoseconds. The muons are similar to electrons ($e^{-}$), except that their mass $m_\mu = 207 m_e$ (where $m_\mu$ and $m_e$ represent the mass of muon and electron, respectively) and that the muon decays into an electron with a half-life of $T_{1/2}=1.52~\mu$s. A continuous beam of negative muons ($\mu ^{-}$) can be selected and transported through a beamline from the target to the experimental area. With the proper optimization of the transport elements (magnetic dipoles, quadrupoles and slits) along the $\pi$E1 beamline, the maximum number of $\mu ^{-}$ particles at a given muon momentum (*p*) and momentum bite ($\Delta p/p$ where $\sigma = \Delta p$) are transported to hit the sample of interest, placed at the target position. Depending on the chosen *p* and the density of the measured sample, the $\mu ^{-}$ penetrate to different depths inside the sample. However the $\mu ^{-}$ doesn’t stop at a precise depth, but is distributed over a range, which is in turn determined by $\Delta p/p$. Immediately after its implantation inside the sample, the $\mu ^{-}$ particles are captured by the atoms present in the sample. This results in the emission of a cascade of muonic X-rays ($\mu$-X rays, or the prompt spectrum) with characteristic energies for each element, until the $\mu ^{-}$ reaches its ground state. The emission of $\mu$-X rays is very similar to the commonly known electronic X-rays (as in X-ray Fluorescence Technique (XRF)), except that the energies of $\mu$-X rays are significantly higher and hence have higher penetration capabilities inside the sample. When the $\mu ^{-}$ particle has reached its ground state, which is very close to the nucleus, there is a strong probability that it will be further captured by the nucleus itself. This leads to a nuclear reaction resulting in the emission of gamma-rays (the delayed spectrum). The resulting $\mu$-X rays and gamma-rays are detected by an appropriate detector setup, arranged around the sample. Further details on the MIXE technique can be found in [1] and [2] .

The detector setup consisted of the MINIBALL Germanium detector array [36] comprising of twenty-six High Purity Germanium (HPGe) detectors, as shown in Fig. 4a. A typical MINIBALL cluster module consists of three Ge detectors (relative efficiency, $\epsilon \sim$60% each) and so twenty-four out of the twenty-six HPGe detectors were MINIBALL detectors. The remaining two HPGe detectors were a low-energy detector and a detector with $\epsilon \sim$70%. This experimental apparatus was originally developed for the muX experiment ([37, 38]). The energy and efficiency calibrations for this setup were performed using the standard radioactive gamma sources, $^{152}$Eu and $^{88}$Y. In addition to the HPGe detectors, we used two scintillator detectors made of polyvinyltoluene (BC-400), placed in vacuum, at the end of the $\pi$E1 beamline: (i) a muon entrance detector of thickness $\sim$200 $\mu$m, to count the number of $\mu ^{-}$ particles and to measure their arrival times and (ii) a 5 mm thick muon veto detector with a 18 mm (diameter) hole. The vacuum inside the $\pi$E1 beamline is separated from air (as the sample is usually placed in air) by a $\sim$10 $\mu$m thick titanium (Ti) window. Figure 4b shows an additional scintillator detector (a large square-shaped detector with a hole) located before the sample (part of the muX setup), placed at $\sim$1 cm away from the Ti window. The signals from this detector were not used for the final analysis and hence are not discussed.

### Measurement of the knob bow fibula (Bügelknopffibel)

**Fig. 5:**
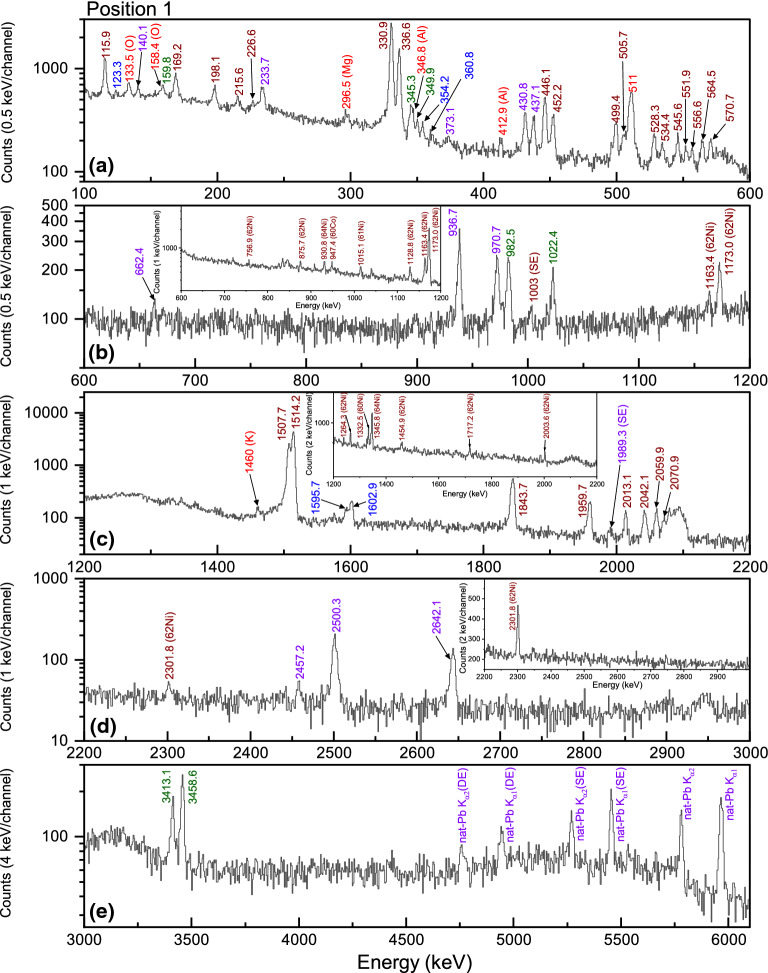
The prompt muonic X-ray ($\mu$-X ray) spectrum from the knob bow fibula, at position 1, in different energy ranges: **a** 100–600  keV, **b** 600–1200 keV, **c** 1200–2200 keV, **d** 2200–3000 keV and **e** 3000–6100 keV. The $\mu$-X rays of copper (Cu), zinc (Zn), tin (Sn) and lead (Pb) are shown in brown, blue, green and purple colors, respectively. The insets in (**b**), (**c**) and (**d**) show the delayed spectrum, which consists of only the gamma rays from $^{60-62,64}$Ni (nickle) and $^{60}$Co (cobalt), as a result of the the capture of muons by the Cu nucleus. These are also marked in brown color. The rest of the contaminants peaks are shown in red

**Fig. 6:**
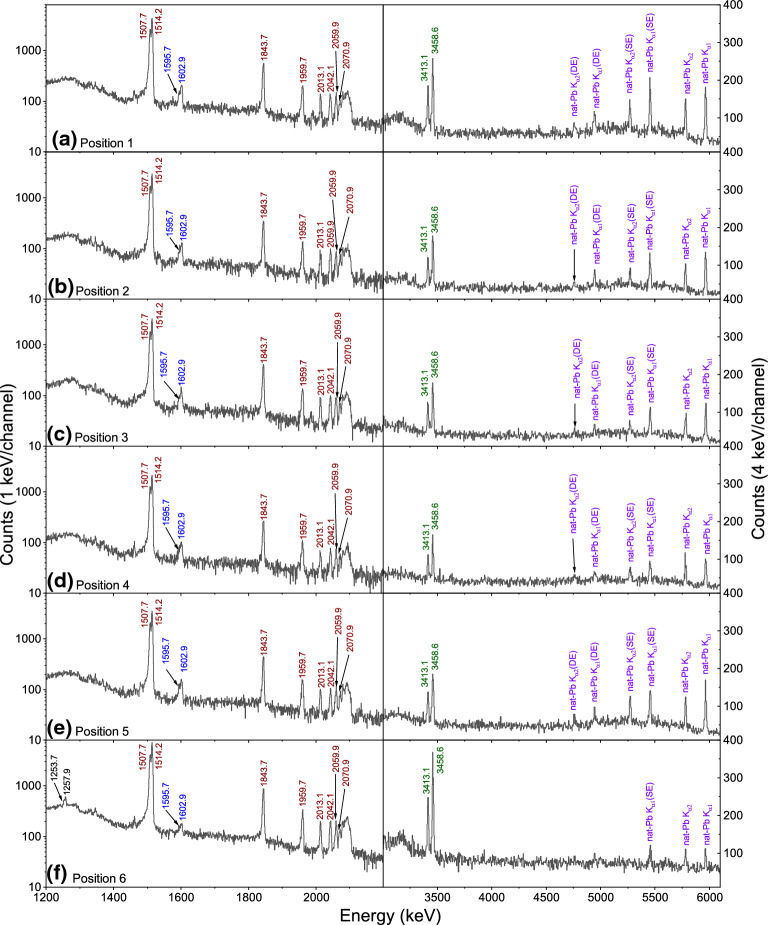
The prompt $\mu$-X ray spectrum from the knob bow fibula at **a** position 1: center of the bow; **b** position 2: side of the bow, not on the spiral side; **c** position 3: side of the bow towards the spiral; **d** position 4: the knob; **e** position 5: the foot; and **f** position 6: the spiral. Two different energy ranges have been shown for each position: 1200 to 2200 keV in the left columns and 3000 to 6100 keV in the right columns. The K-series lines of Cu, Zn, Sn, Pb and Fe are shown in brown, blue, green, purple, and black colors, respectively. The Fe signal is seen only in position 6

The knob bow fibula (Bügelknopffibel) was placed at $\sim$9 cm after the Ti window, supported on cut out polyethylene foam and placed on an iron stand. A 1 cm thick polyethylene (PE) plastic collimator with a 3 mm diameter hole, as shown in Fig. 4b, was placed between the fibula and the additional scintillator detector, to direct the $\mu ^{-}$ beam at a particular position on the fibula. Six positions were chosen for the present study: (1) center of the bow, (2) side of the bow, not on the spiral side, (3) side of the bow towards the spiral, (4) the knob, (5) the foot, and (6) the spiral. These positions are marked in Fig. 4c. The measurement at all these six positions was performed with a $\mu ^{-}$ beam at $p~=$ 33 MeV/c with $\Delta p/p$ = 2%. This corresponds to a stopping depth of $320\pm 20$ $\mu$m inside a pure Cu sample. Each position of the fibula was irradiated with muons for $\sim$1.5 hours.

The detailed prompt $\mu$-X ray and delayed gamma-ray spectra, for position 1 at different energy ranges, are shown in Fig. 5. From this figure, it is evident that the base element is copper (Cu), as one can see all the K, L, M, and N series $\mu$-X rays (shown in brown color). This indicates that the fibula is made of Cu alloy. The $\mu$-X rays from zinc (Zn), tin (Sn), and lead (Pb) are shown in blue, green and purple colors, respectively, which appear to be the major components of the Cu alloy. The $\mu$-X ray energies, as shown in the Fig. 5, for these elements are taken from [39] and [40] database. The K-series $\mu$-X rays of Pb are written as nat-Pb (natural-Pb), in the Fig. 5e, because Pb exists in nature as four stable isotopes $^{204,206,207,208}$Pb [41] which have slightly different energies. Some of the $\mu$-X rays in Fig. 5 are marked as “Single Escape (SE)” or “Double Escape (DE)”, which occur due to the interaction of the high-energy $\mu$-X rays (>1022 keV) with the HPGe detector [42]. Below 100 keV, there were very high amplitude $\mu$-X ray peaks from carbon (C), not shown in this figure. These peaks arise as a large fraction of the $\mu ^{-}$ beam stops inside the PE collimator and only those which make it through the hole, stop inside the fibula. The inset in Fig. 5b–d show the delayed gamma-ray spectrum. As can be seen from these spectra, all the gamma rays are emitted from $^{60-64}$Ni (nickle) and $^{60}$Co (cobalt), as a result of muon capture by Cu nuclei. The energy of the gamma rays were taken from the [41] webpage.

Apart from the above-mentioned major components, we also see (in Fig. 5a,c) the (i) $\mu$-X rays of oxygen (O), aluminium (Al), and magnesium (Mg); (ii) the 1460 keV gamma ray from the decay of $^{40}$K (potassium); and (iii) the 511 keV positron-electron annihilation peak (all of these are shown in red colors). The Mg, Al, K, and O are believed to be elements from the soil, from where the fibula was excavated, or to be undeliberate mixing in the alloy from the ore or manufacturing process. No prior soil analysis (from the excavated site) was carried out and hence it cannot be confirmed. Usually Mg, Al and potassium carbonate (K$_2$CO$_3$) are present in soil and during long term soil-storage, the Cu alloy gets corroded and the soil components can go very deep inside the material. As the corrosion process of Cu alloy depends on many factors such as relative humidity, soil pH, ions present in soil, soil aeration, etc. and since no prior documentation exists on the soil from the excavated area, we do not delve deeper into the origin of these contamination peaks.

Figure 6 shows the $\mu$-X ray spectra from all the six different positions on the fibula, at two different energy ranges focusing only on the K-series lines. In the first five positions (Fig. 6a–e), the $\mu$-X rays for Cu, Zn, Sn, and Pb are observed, which are depicted in brown, blue, green, and purple colors, respectively (same as in Fig. 5). In position 6 (Fig. 6f), apart from the $\mu$-X rays of Cu, Zn, Sn, and Pb, the $\mu$-X ray of iron (Fe) was observed, shown in black color. The observation of Fe signal at position 6 is verified by the presence of iron (hydr)oxides (corrosion product, as seen in Fig. 1b,c and Fig. 3(bottom)) arising due to the fact that the axis-pin is made of Fe.

### Measurement of the Certified Reference Materials (CRMs)

In order to obtain the elemental composition of the fibula, a few Certified Reference Materials (CRMs) of brass and bronze were measured with the same experimental setup. Similar experiments, with CRMs, were performed in the pulsed muon beam facilities at ISIS, Rutherford Appleton Laboratory (RAL), United Kingdom [6, 43] and Japan Proton Accelerator Research Complex, Muon Science Establishment (J-PARC-MUSE) [4]. Hillier et al. [43] has used the ratio of the intensities of the $\mu$-X ray of an element to that of Cu, in bronze CRM, to directly compare with the certified composition. However, in [6], a calibration curve was first obtained for copper alloy CRMs, by plotting the weight percentage (wt%) of an element versus the experimentally observed relative intensity of the $\mu$-X ray of that element, and did not use the ratios. This calibration curve was then used to determine the elemental composition of archaeological bronze samples. The approach used by [6] is similar to the approach used in Energy Dispersive X-Ray Fluorescence (EDXRF) [44], where the calibration curve is obtained by plotting the counts per second (cps) under the X-ray peak of an element versus the percentage composition of the same element in the CRM. A calibration curve with the ratio of the intensities of the $\mu$-X ray of an element to that of Cu, in bronze CRM, versus the ratio of the wt% of the element to that of Cu was used in [4] to determine the unknown elemental composition of archaeological samples. Since the above references use different methods to obtain the elemental compositions, we have used three different methods to see how well each method works: (i) Method 1: the intensity of the $\mu$-X ray of an element normalized to the muon entrance counts is compared with the atomic percentage (at%) of the element to obtain a calibration curve; (ii) Method 2: the ratio of the intensities of the $\mu$-X ray of an element to that of Cu, in bronze and brass CRMs, is compared to the ratio of the at% of the element to that of Cu to obtain the calibration curve; and (iii) Method 3: the ratio of the intensities of the $\mu$-X ray of an element to that of Cu is corrected for the detector efficiency, branching ratio and the muon capture probability to directly obtain the ratio of at% of the element and Cu. The method 3 is a powerful method as it can be used to determine the elemental composition of an unknown sample without the use of CRMs.

The different brass and bronze standards were produced by the Analytical Reference Material International (ARMI|MBH) [45] and Brammer Standard Company (BS) [46]. The ARMI|MBH Brass CRMs, measured with the MIXE technique, are 31X B18 (batch K) and 31X B22 (batch F). The sizes of all the aforementioned samples were $\sim$45.5 mm in diameter and $\sim$17.5 mm thick. The bronze CRMs measured during this experiment are 32X SN4 (batch B) (from ARMI|MBH), IARM 184A (from ARMI|MBH) and BS938-1 (from BS). The sizes of the bronze CRMs are 45.7 mm diameter, 18.0 mm thick; 37.5 mm diameter, 14.9 mm thick; and 41.2 mm diameter, 11.9 mm thick, respectively. The certificates of the various CRMs, used in this experiment, can be obtained from the above-mentioned references. Since the uncertainties on the wt%s given in these certificates are based on a confidence interval of 95%, a factor of 1.96 was divided from these numbers in order to convert to 1 standard deviation (S.D.). Contrary to the fibula measurements, the PE collimator was not used and the CRMs were placed at a distance of $\sim$17.7 cm from the Ti window. Similar to the fibula, the measurement of the CRMs were performed with the $\mu ^{-}$ beam at $p~=$ 33 MeV/c with $\Delta p/p$ = 2%. The brass and the bronze CRMs were irradiated with muons for $\sim$0.5 and 1.5 h, respectively.

The third and fourth columns in Table 1 shows the list of elements and their wt%, as given in the certificates of the above-mentioned CRMs. Only those elements that were observed in the current experiment are shown in this table. The rest of the elements which were not observed, but mentioned in the certificates, are summed and written as “others” in the table. Since the $\mu ^{-}$ interacts with the atom, it is more appropriate to correlate the results with the at%. Hence, the wt% were first converted to at% (in the sixth column of Table 1) using the following equation (assuming an alloy consisting of elements $X_1$, $X_2$, ..., $X_k$, ..., $X_n$):

1 $$\begin{aligned} at_{X_k}\% = \frac{wt_{X_k}\%/at. wt_{X_k}}{\sum \limits _{i=X_1}^{i=X_n} wt_{i}\%/at. wt_{i}} \times 100 \end{aligned}$$

where $at_{X_k}\%$ represents the at% of the element $X_k$, $wt_{X_k}\%$ represents the wt% of the element $X_k$, and the $at. wt_{X_k}$ is the atomic weight of the element $X_k$. The atomic weights, along with their errors, were taken from the [47] webpage. The elements with wt% lower than $\sim$0.4% were not observed with the present setup.

**Table 1:** Measured CRMs with the list of elements, their weight percentages (wt%), the ratio of wt% of an element X (X: Zn, Sn, Pb) and the wt% of Cu $\left( \frac {wt\%_{X}}{wt\%_{Cu}}\right)$, the atomic percentage (at%), the ratio of at% of an element X (X: Zn, Sn, Pb) and the at% of Cu $\left( \frac {at\%_{X}}{at\%_{Cu}}\right)$, the normalized intensity ($I_{norm}$), the ratio of the intensities of element X wrt Cu $( \frac {I_{X}}{I_{Cu}})$, and ratio of intensities corrected for the efficiency, muon capture probability and the branching ratio $( \frac {I_{X, \epsilon , cap, br}}{I_{Cu, \epsilon , cap, br}})$

Alloy	Name	Element	wt%	$\frac{wt\%_{X}}{wt\%_{Cu}}$	at%	$\frac{at\%_{X}}{at\%_{Cu}}$	$I_{norm} \times 10^4$	$\frac{I_{X}}{I_{Cu}}$	$\frac{I_{X, \epsilon , cap, br}}{I_{Cu, \epsilon , cap, br}}$
Brass	31X B18 K	Cu	59.37 ± 0.05		60.41 ± 0.06		64.6 ± 0.3		
		Zn	39.41 ± 0.04	0.6638 ± 0.0008	38.97 ± 0.04	0.645 ± 0.001	43.3 ± 0.3	0.670 ± 0.005	0.64 ± 0.08
		Pb	1.018 ± 0.005	0.01715 ± 0.00008	0.318 ± 0.001	0.00526 ± 0.00002	0.30 ± 0.03	0.0047 ± 0.0004	
		others	0.270 ± 0.002		0.300 ± 0.002				
	31X B22 F	Cu	82.47 ± 0.06		82.83 ± 0.10		89.8 ± 0.4		
		Zn	15.92 ± 0.07	0.1930 ± 0.0008	15.54 ± 0.07	0.1876 ± 0.0008	17.1 ± 0.2	0.190 ± 0.003	0.18 ± 0.02
		others	1.599 ± 0.008		1.628 ± 0.008				
Bronze	32X SN4 B	Cu	76.87 ± 0.06		83.79 ± 0.08		91.6 ± 0.2		
		Sn	18.96 ± 0.03	0.2466 ± 0.0004	11.06 ± 0.02	0.1320 ± 0.0003	7.46 ± 0.08	0.0814 ± 0.0009	
		P	1.208 ± 0.009		2.701 ± 0.019				
		Pb	0.864 ± 0.004	0.01124 ± 0.00005	0.289 ± 0.001	0.00345 ± 0.00001	0.23 ± 0.02	0.0026 ± 0.0002	
		Ni	0.607 ± 0.004		0.716 ± 0.004				
		Zn	0.496 ± 0.004	0.00645 ± 0.00005	0.525 ± 0.004	0.00627 ± 0.00004	0.58 ± 0.05	0.0063 ± 0.0005	
		Ag	0.495 ± 0.004		0.318 ± 0.003				
		others	0.506 ± 0.002		0.598 ± 0.002				
	IARM 184A	Cu	74		88		84.9 ± 0.2		
		Pb	19.0 ± 0.2	0.257 ± 0.002	6.94 ± 0.06	0.0787 ± 0.0006	4.12 ± 0.04	0.0485 ± 0.0005	
		Sn	6.00 ± 0.05	0.0811 ± 0.0007	3.82 ± 0.03	0.0434 ± 0.0004	2.21 ± 0.04	0.0261 ± 0.0005	
		Zn	0.37 ± 0.02	0.0050 ± 0.0002	0.43 ± 0.02	0.0048 ± 0.0002	0.66 ± 0.03	0.0078 ± 0.0004	
		others	0.664 ± 0.010		0.696 ± 0.009				
	BS 938-1	Cu	77.10 ± 0.08		89.18 ± 0.12		88.2 ± 0.2		
		Pb	14.80 ± 0.05	0.1920 ± 0.0007	5.25 ± 0.02	0.0589 ± 0.0002	3.14 ± 0.03	0.0356 ± 0.0004	
		Sn	7.16 ± 0.03	0.0929 ± 0.0003	4.43 ± 0.02	0.0497 ± 0.0002	2.70 ± 0.04	0.0306 ± 0.0004	
		Ni	0.490 ± 0.005		0.614 ± 0.006				
		others	0.392 ± 0.005		0.517 ± 0.006				

Uncertainties are 1 standard deviation (S.D.)

### Experimental results

#### CRMs

**Fig. 7:**
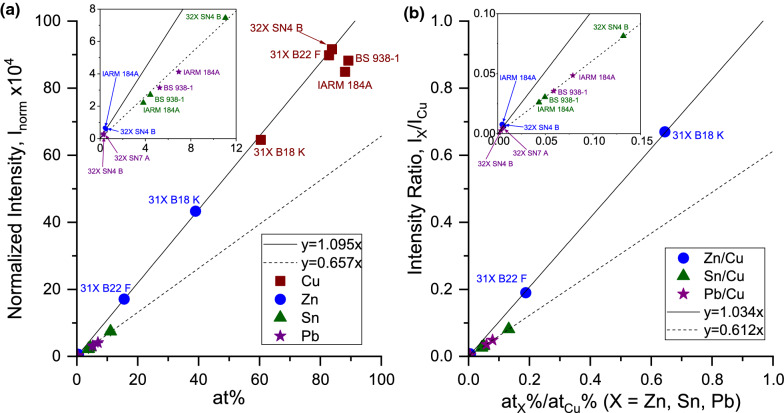
Calibration curve for the brass and bronze CRMs: **a** Method 1: plot of intensity of the $\mu$-X rays of the different elements (Cu, Zn, Sn, and Pb), normalized with the number of muons vs. the at% of the elements; **b** Method 2: plot of the ratio of intensity of the $\mu$-X rays of the different elements (Zn, Sn, and Pb) and that of of Cu *vs.* the ratio of the at% of the elements with respect to Cu. The insets in (**a**) and (**b**) show the enlarged plots of the corresponding figures in order to emphasize the Sn and Pb data points

*Method 1:* For the subsequent analysis, the following $\mu$-X ray lines were considered: (i) $K_{\alpha 1}$ and $K_{\alpha 2}$ lines for Cu (1507.7 and 1514.2 keV), (ii) $K_{\alpha 1}$ and $K_{\alpha 2}$ lines for Zn (1595.7 and 1602.9 keV), (iii) $L_{\alpha }$ line of Sn (982.5 keV) and (iv) $L_{\alpha }$ line of Pb (2500.3 keV). In this first method, the area under these peaks (for the different elements) was evaluated and then divided with the total number of muons in the muon entrance counter to determine the experimental data points, as shown in Fig 7a. The experimental data points for Cu, Zn, Sn, and Pb are shown in brown filled square, blue filled circle, green filled triangle, and purple filled star symbols, respectively. The data points, used to generate this plot, are shown in the eighth column of Table 1. Two linear polynomials $y~=~1.095\times x$ and $y~=~0.657\times x$ are used to fit these data points. It can be seen from this plot that the experimental data points for Cu and Zn lie on the line $y~=~1.095\times x$, whereas those for Sn and Pb lie on the line $y~=~0.657\times x$. As can be seen from the table and the figure, two data points of Cu (corresponding to IARM 184A and BS 938-1) do not follow the linear relationship. The reason is that the diameter of the IARM 184A and BS 938-1 is smaller than the diameters of the other samples, and hence not all of the muons that make it through the muon entrance counter collide with the above-mentioned two samples. The inset in Fig. 7a shows the enlarged plot of the same figure in order to emphasize the Sn and Pb data points. The effect of the sizes of IARM 184A and BS 938-1, which was very evident in Cu, is not so evident for Sn and Pb, probably because the at% of these elements is very small. But still, one can see from this inset that the Sn and Pb points for these two samples are slightly lower than the rest.

The other important thing to note is whether one should use the wt% or the at% for such comparisons. As can be seen from both the table, the linear relationship between the normalized intensity works for at% and not for wt%. This can be explained easily for the Cu case: The wt% of Cu in 31X B22 F is 82.47 ± 0.06%, while the at% is 82.83 ± 0.10%. The wt% of Cu in 32X SN4 B is 76.87 ± 0.06%, while the at% is 83.79 ± 0.08%. This indicates that although the wt% decreases from 31X B22 F to 32X SN4 B, the at% has in fact increased. Indeed, the experimental normalized intensity plot shows an increasing trend from 31X B22 F to 32X SN4 B emphasizing that one should compare such ratios with the at% and not the wt%. However, for the rest of the elements, the trend of wt% and at% seems to be the same.

*Method 2:* In the second method, the same $\mu$-X ray peaks, as described in method 1, are considered. The area under these peaks, for the different elements were first evaluated, same as in method 1. Now, instead of dividing these areas by the muon entrance counts, the areas for the elements Zn, Sn and Pb were divided to those of Cu. These experimentally determined points are shown in the ninth column of Table 1 and are represented on the y-axis in Fig. 7b. The x-axis in Fig. 7b shows the ratio of the at% of the elements with that of Cu (also shown in the seventh column in Table 1). The data points for the ratios Zn/Cu, Sn/Cu, and Pb/Cu are shown as blue filled circles, green filled triangles, and purple filled stars, respectively. Two linear polynomials $y~=~1.034\times x$ and $y~=~0.612\times x$ are used to fit these data points. It can be seen from this plot that the experimental data points for Zn/Cu lie on the line $y~=~1.034\times x$, whereas those for Sn/Cu and Pb/Cu lie on the line $y~=~0.612\times x$, which is very similar to method 1. The discrepancy in the numbers, due to the different diameters of the IARM 184A and BS938-1, as seen in method 1, no longer has an effect in this plot because using the ratios cancels out such effects. The inset in Fig. 7b shows the zoomed plot of the same figure in order to emphasize the Sn and Pb data points. As can be seen here, in comparison to method 1, all the data points lie perfectly on the linear fits.

For all the CRMs considered here, the trend of wt%$_{X}$/wt%$_{Cu}$ and at%$_{X}$/at%$_{Cu}$ (X = Zn, Sn, and Pb) is the same, and hence it is difficult to establish if the usage of ratios of wt% or at% is correct. In the current manuscript, we have preferred to use the ratios of at%, instead of ratios of wt%, similar to method 1.

*Method 3:* In the third method, the following $\mu$-X ray lines were considered: (i) $K_{\alpha 1}$ and $K_{\alpha 2}$ lines for Cu (1507.7 and 1514.2 keV), (ii) $K_{\alpha 1}$ and $K_{\alpha 2}$ lines for Zn (1595.7 and 1602.9 keV). The area under these peaks, for the different elements were first evaluated, same as in methods 1 and 2. Then these areas were divided by the efficiency ($\epsilon$) of the setup at that energy, the muon capture probability (*cap*), and the branching ratio (*br*) of the $K_{\alpha }$ transitions to get the corrected intensities. The muon capture probability of the different transitions were obtained from [48]. The branching ratio of the *K*-series transitions were determined from the pure sample of Cu which was also measured with the same experimental setup. However since we did not measure any pure sample of Zn, the *K*-series branching ratio was determined from the CRM alloy itself. Then the ratio of the corrected intensities for Zn was divided by that of Cu to get the experimental data points, as shown in the last column of Table 1. As can be seen from these values, they agree well with the ratio of the at%, showing the advantage of using this method over other methods, without the need of measuring any CRMs. However, the errors for the muon capture probabilities in [48] are very high and this results in huge errors for the intensity ratios. So, this indicates the need to re-measure the muon capture probabilities with greater precision. This analysis could also be applied for the other elements Sn and Pb (by considering the K-series transitions $K_{\alpha 1}$ and $K_{\alpha 2}$ lines of Sn (3413.1 and 3458.6 keV) and $K_{\alpha 1}$ and $K_{\alpha 2}$ lines of Pb (5784.5 and 5970.8 keV)) but this was not done owing to the large errors in muon capture probabilities.

#### Knob bow fibula

The method 1 was not used to determine the elemental composition of the fibula because a collimator was used in this case and hence not all the muons that pass through the muon entrance counter reach the fibula. Method 3 requires the usage of the muon capture probabilities, but owing to the large error bars, this has not been used in the present work. So, only the method 2 has been utilized to determine the elemental composition of the fibula.

With the method 2, the same peaks as in the case of CRMs, were used to calculate the areas under the peaks of the different elements Cu, Zn, Sn and Pb. The areas of Zn, Sn and Pb were then divided by that of Cu to get the intensity ratios, which are shown in the third column of Table 2. These ratios are then divided by the calibration factors of 1.034 (for Zn) and 0.612 (for Sn and Pb), as deduced from the method 2 in Sect. "CRMs". This results in the conversion from the experimental data to the ratio of the at% with that of Cu. It is assumed that the fibula under question is made of Cu, Zn, Sn, and Pb because the spectra in Figs. 5 and 6 show that these are the main elements and the others are adjacent elements and/or contaminants. The Fe peak at position 6 is due to the corrosion products from the axis-pin and not the spiral  itself and so it can be excluded from the analysis. Under this assumption, one can then conclude that the contributions from Cu, Zn, Sn, and Pb amounts to 100%. From the experiment we know the ratios of at% Zn/Cu = x1, Sn/Cu = x2, and Pb/Cu = x3. Using the following equations,

2 $$\begin{aligned}{} & {} at_{Cu}\% + at_{Zn}\% + at_{Sn}\% + at_{Pb}\% = 100\% \end{aligned}$$

3 $$\begin{aligned}{} & {} at_{Cu}\% + x1\times at_{Cu}\% + x2\times at_{Cu}\% + x3\times at_{Cu}\% = 100\% \end{aligned}$$

4 $$\begin{aligned}{} & {} at_{Cu}\% = \frac{100\%}{1 + x1+ x2 + x3} \end{aligned}$$

(5)

one can calculate the at% of all the individual elements, which are shown in the fourth column of Table 2. These at% can then be converted back to wt% by using the following formula (assuming an alloy consisting of elements $X_1$, $X_2$, ..., $X_k$, ..., $X_n$):

6 $$\begin{aligned} wt_{X_k}\% = \frac{at_{X_k}\%\times at. wt_{X_k}}{\sum \limits _{i=X_1}^{i=X_n} at_{i}\% \times at. wt_{i}} \times 100 \end{aligned}$$

**Fig. 8:**
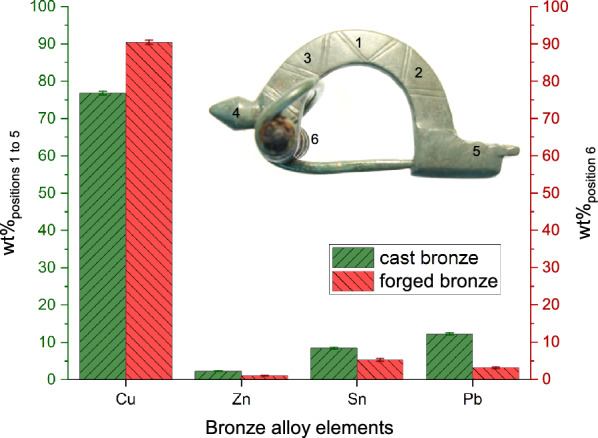
Bar graph (with error bars) showing the (i) weighted average of the wt% of positions 1 to 5 in green (with up-sloping hatches) and (ii) wt% of position 6 in red (with down-sloping hatches). The picture of the knob bow fibula, with the different positions marked, is shown in the inset

The calculated wt% of the different elements are shown in the fifth column of Table 2. A notable feature is the variation of wt% of Pb in the different parts of the fibula: (i) the knob with 13.0 ± 0.6 wt% Pb (position 4), bow 11.9 ± 0.4 (weighted average of positions 1, 2, and 3) and foot 12.5 ± 0.9 (position 5) show a higher Pb content while (ii) the spiral of the fibula with 3.2 ± 0.2 wt% Pb (position 6) has a significantly lower Pb content. The numbers in this table show that the elemental composition of the fibula is the same in position 1 to position 5, but different in position 6. Since positions 1 to 5 have the same composition, one can calculate the weighted average of the wt%s for these positions, resulting in 76.8 ± 0.5%, 2.3 ± 0.1%, 8.5 ± 0.2%, and 12.3 ± 0.3% for Cu, Zn, Sn, and Pb, respectively. A visual depiction of the weighted average of the wt%s for position 1 to 5 (wt%$_{\mathrm {positions~1~to~5}}$) and the wt% for position 6 (wt%$_{\mathrm {position~6}}$) for all the bronze alloy elements, in Table 2, is shown in Fig. 8 with green (with up-sloping hatches) and red (with down-sloping hatches) colors, respectively.

**Table 2:** Table showing the ratio of the intensities of element X wrt Cu $( \frac {I_{X}}{I_{Cu}})$ (X: Zn, Sn, Pb), the atomic percentage (at%) and the weight percentage (wt%) for the knob bow fibula at six different positions

Position No.	Element	$I_{X}/I_{Cu}$	at%	wt%
Position 1	Cu		87.8 ± 0.6	76.7 ± 0.9
	Zn	0.030 ± 0.002	2.5 ± 0.2	2.2 ± 0.2
	Sn	0.037 ± 0.002	5.4 ± 0.3	8.8 ± 0.5
	**Pb**	0.030 ± 0.002	4.3 ± 0.2	**12.2 ± 0.6**
Position 2	Cu		87.9 ± 0.8	76.4 ± 1.3
	Zn	0.030 ± 0.003	2.5 ± 0.2	2.2 ± 0.2
	Sn	0.033 ± 0.002	4.8 ± 0.4	7.8 ± 0.7
	**Pb**	0.034 ± 0.002	4.8 ± 0.4	**13.6 ± 1.2**
Position 3	Cu		89.1 ± 0.7	78.8 ± 1.0
	Zn	0.026 ± 0.003	2.2 ± 0.2	2.0 ± 0.2
	Sn	0.033 ± 0.002	4.8 ± 0.3	7.9 ± 0.5
	**Pb**	0.027 ± 0.002	3.9 ± 0.2	**11.2 ± 0.6**
Position 4	Cu		86.6 ± 0.9	75.1 ± 1.2
	Zn	0.038 ± 0.005	3.2 ± 0.4	2.8 ± 0.4
	Sn	0.040 ± 0.003	5.6 ± 0.4	9.1 ± 0.6
	**Pb**	0.032 ± 0.002	4.6 ± 0.2	**13.0 ± 0.6**
Position 5	Cu		87.2 ± 0.8	76.1 ± 1.2
	Zn	0.037 ± 0.004	3.1 ± 0.3	2.8 ± 0.3
	Sn	0.037 ± 0.002	5.3 ± 0.3	8.6 ± 0.5
	**Pb**	0.031 ± 0.002	4.4 ± 0.3	**12.5 ± 0.9**
Position 6	Cu		94.8 ± 0.4	90.4 ± 0.6
	Zn	0.012 ± 0.001	1.1 ± 0.1	1.1 ± 0.1
	Sn	0.020 ± 0.001	3.0 ± 0.2	5.3 ± 0.4
	**Pb**	0.0066 ± 0.0005	1.03 ± 0.07	**3.2 ± 0.2**

The lead content has been highlighted in bold, as it is most decisive for the material properties. Uncertainties are 1 SD

## Discussion

The fibula is in a good state of preservation. By optical analysis it can be assumed that a noble patina out of Cuprite (Cu$_2$O) and Malachite (Cu$_2$CO$_3$(OH)$_2$) has formed. The thickness of this passivating corrosion layer is typically between 10 and 100 $\mu$m [49, pp. 241–242]. With a measurement depth of 0.3–0.4 mm, a bulk measurement below the corrosion layer is probable if the formation of a noble patina is assumed. However, it was not possible to analyze the corrosion layer, since this could only be done in a destructive manner. It should be noted that corrosion layer thicknesses on archaeological objects made of copper alloys can have very different characteristics and form correspondingly thick layers of 1–2 mm [50, p. 7]. Fibulae have been investigated by metal analysis many times in the last decades. Non-destructive methods such as X-Ray Fluorescence (XRF) and methods that require sampling such as Atomic Absorption Spectroscopy (AAS) and Scanning Electron Microscopy with Energy Dispersive X-ray analysis (SEM-EDX) have been used. A large number of provincial Roman and Germanic objects—among them 4 knob bow brooches—could be evaluated metallographically and elementally within the project “Römische und germanische Bunt- und Edelmetallfunde im Vergleich. Archäometallurgische Untersuchungen ausgehend von elbgermanischen Körpergräbern” [10, pp. 107–381]. A small selection of publications with analysis results of Imperial Period non-ferrous and precious metal objects can be found, for example, in [51, Table 1]. It shows that different alloys were used for different groups of objects. Fibulae represent a special group in this context, since different manufacturing methods and alloy types are often found on the same piece: the spiral should be elastic-bendable, while the bow should be stable and ornamental. (Late) antique copper alloys always contained the specific admixture of tin, zinc and lead. Depending on the application, one or more of the elements were used in different concentrations. Other trace elements, due to the ore used, the use of recycled material, or soil storage, will not be considered here. Similarly, the iron measured by the corrosion products on the spiral (position 6) was not considered in the copper alloy calculations (see Sect. Knob bow fibula). The casting properties of the copper alloys are influenced depending on the type of admixture(s). For example, the addition of tin and/or lead significantly reduces the melting temperature of the alloy due to the lower melting points. A high lead content is well suited for casting objects due to better flow properties. Whereas an object with a low lead content can also be forged well. The copper alloys of antiquity could contain zinc contents of up to 30% (aurichalcum). Tin content was usually less than 15% and lead could be as high as about 25% [52, p. 14]. The positions 1-5 measured on the knob bow fibula show the same elemental composition (in wt%) with approximately 77% copper, 2% zinc, 8% tin, and 12% lead (see Fig. 8). This alloy is thus ideally suited for casting. The zinc content of about 2% serves as a deoxidizer in the alloy and increases flowability for bubble-free results. The relatively high lead content favourably lowers the melting point and reduces the viscosity for better flow of the metal into the mould. Thus, according to [10, p. 278, Tab. 30], the alloy can also be characterized as cast bronze (Alloy 4) with a very low melting range from 954 °C down to 327 °C. Due to the measurement inaccuracies (lower precision), it is not possible to trace a more differentiated statement on the element distribution within the fibula corpus. However, a different element distribution in such small objects is also not to be expected. The orientation of the dendrites in the metallographic section could indicate how or in which mould the piece was cast, but this is a destructive analysis method, which was not intended or could be avoided in the case of this fibula. Position 6 shows a higher copper with lower lead content (see Fig. 8), with a composition (in wt%) of approximately 90% copper, 1% zinc, 5% tin, and 3% lead, which also allowed the piece to be forged and cold worked (see manufacturing traces in Fig. 3). There is an ongoing debate about the maximum level of lead content suitable for forging [52, pp. 15–16]. A low lead content is advantageous in any case, since up to a certain level the machinability of the workpiece can be improved. With a higher lead content, the workpiece loses strength and ductility. For the mechanically stressed workpiece of the spiral and needle, the element composition shown at position 6 seems ideal and can therefore also be characterized as forging bronze (Alloy 2) according to [10]. The original color of the bronze object can be reconstructed based on the element composition. It must have been a brownish shiny fibula. As the extensive study on Roman and Germanic non-ferrous and precious metals [10] impressively shows, Roman as well as Germanic craftsmen selected their source material consciously and by variety. If one compares the alloys of individual knob bow brooches, it becomes clear that they are by no means uniform. Therefore, decentralized, independent fabrications can be assumed and furthermore recycling and individual mixing of the source metals can be concluded. The simultaneous different types of alloys show that there was no centralized control of the manufacturing methods in the empire, but that local workshops worked with their own raw materials.

## Conclusions

The knob bow fibula (*Bügelknopffibel*) 2018.005.G07151 of the Germanic type *Leutkirch* was measured for the elemental composition at six different positions. Positions 1–5 represent different points of the same workpiece with identical elemental composition (in wt%) of approx. 77 % copper, 2 % zinc, 8 % tin and 12 % lead. This type of alloy is ideal for a casting. A casting is always preferred when attention is paid to surface details and ornamentation. In the case of the fibula, this is exactly the case: the bow is the decorative element of the fibula. Position 6 shows the elemental composition of the spiral together with corrosion products of the axis-pin. This alloy with approx. 90 % copper, 1 % zinc, 5 % tin and 3 % lead (in wt%) has a high strength and ductility and is therefore ideally suited for the workpiece of the needle, which is subject to extensive mechanical stress during use. The analysis once again impressively shows that the ancient craftsmen deliberately selected and used their materials quite precisely to suit their purposes.

The measurements belong to the first group of objects analyzed with the Muon-Induced X-ray Emission technique (MIXE) at Paul Scherrer Institute (PSI) and therefore served to validate the measurement method. Elemental (and isotopic) analysis of archaeological objects with MIXE is still in its infancy. Both the measurement setup and the data analysis are continuously being refined. In [11], the detection limit was estimated to be 0.81 wt% with $\sim$10 hours of measurement time and [6] gave a sensitivity of 1% with 12–18 h of irradiation time. In the present work, the detection limit is 0.4 wt% with $\sim$1.5 hours of measurement time. A significant increase in measurement accuracy is still being sought. In addition, extensive simulation of the complicated geometry of archaeological objects is necessary in order to determine the attenuation of the muonic X-rays inside the archaeological material of interest (not as high as electronic X-rays, but certainly not completely negligible) for the proper determination of the elemental composition. Nevertheless, we along with the other published works so far [4, 6–9, 11–13] have demonstrated that MIXE is a very promising new method for completely non-destructive quantitative elemental analysis of valuable objects such as archaeological cultural artefacts. An immense advantage is that, due to the high-energy muonic X-rays, the analysis technique allows measurements up to several centimetres deep—depending on the density of the sample—and thus, if necessary, measurements can be made below the corrosion layer, coatings or surface enrichments. The measurement results show that a completely non-destructive element analysis below the surface is possible.

## Data Availability

The data and materials supporting the conclusions of this article are available from the corresponding author on reasonable request.

## Abbreviations

MIXE: Muon Induced X-ray Emission
PSI: Paul Scherrer Institute
NAA: Neutron activation analysis
XRF: X-ray fluorescence
HPGe: High purity germanium
PE: Polyethylene
SE: Single escape
DE: Double escape
CRM: Certified reference material
RAL: Rutherford Appleton Laboratory
J-PARC-MUSE: Japan Proton Accelerator Research Complex, Muon Science Establishment
EDXRF: Energy Dispersive X-Ray Fluorescence
ARMI: Analytical Reference Material International
BS: Brammer Standard
AAS: Atomic Absorption Spectroscopy
SD: Standard deviation
Cu: Copper
Zn: Zinc
Sn: Tin
Pb: Lead
Ni: Nickle
Co: Cobalt
O: Oxygen
Mg: Magnesium
Al: Aluminium
K: Potassium
Ti: Titanium
C: Carbon
HIPA: High Intensity Proton Accelerator
Fe: Iron
Cps: Counts per second
SEM-EDX: Scanning Electron Microscopy with Energy Dispersive X-ray

## Footnotes

1 This technique is not uniformly named in the literature. In addition to the acronym MIXE [1–4], other acronyms like MXA (Muonic X-ray Analysis) [5], MAXRS (Muonic Atom X-ray Spectroscopy) [6] and $\mu$XES (Muonic X-ray Emission Spectroscopy) [7–9] have been used so far.
